# Proteinopathies as Hallmarks of Impaired Gene Expression, Proteostasis and Mitochondrial Function in Amyotrophic Lateral Sclerosis

**DOI:** 10.3389/fnins.2021.783624

**Published:** 2021-12-23

**Authors:** Bridget C. Benson, Pamela J. Shaw, Mimoun Azzouz, J. Robin Highley, Guillaume M. Hautbergue

**Affiliations:** ^1^Department of Neuroscience, Sheffield Institute for Translational Neuroscience (SITraN), University of Sheffield, Sheffield, United Kingdom; ^2^Neuroscience Institute, University of Sheffield, Sheffield, United Kingdom; ^3^Healthy Lifespan Institute (HELSI), University of Sheffield, Sheffield, United Kingdom

**Keywords:** proteinopathies, RNA metabolism alteration, mitochondrial dysfunction, impaired proteostasis, amyotrophic lateral sclerosis

## Abstract

Amyotrophic lateral sclerosis (ALS) is a fatal adult-onset neurodegenerative disease characterized by progressive degeneration of upper and lower motor neurons. As with the majority of neurodegenerative diseases, the pathological hallmarks of ALS involve proteinopathies which lead to the formation of various polyubiquitylated protein aggregates in neurons and glia. ALS is a highly heterogeneous disease, with both familial and sporadic forms arising from the convergence of multiple disease mechanisms, many of which remain elusive. There has been considerable research effort invested into exploring these disease mechanisms and in recent years dysregulation of RNA metabolism and mitochondrial function have emerged as of crucial importance to the onset and development of ALS proteinopathies. Widespread alterations of the RNA metabolism and post-translational processing of proteins lead to the disruption of multiple biological pathways. Abnormal mitochondrial structure, impaired ATP production, dysregulation of energy metabolism and calcium homeostasis as well as apoptosis have been implicated in the neurodegenerative process. Dysfunctional mitochondria further accumulate in ALS motor neurons and reflect a wider failure of cellular quality control systems, including mitophagy and other autophagic processes. Here, we review the evidence for RNA and mitochondrial dysfunction as some of the earliest critical pathophysiological events leading to the development of ALS proteinopathies, explore their relative pathological contributions and their points of convergence with other key disease mechanisms. This review will focus primarily on mutations in genes causing four major types of ALS (*C9ORF72, SOD1, TARDBP/*TDP-43, and *FUS*) and in protein homeostasis genes (*SQSTM1, OPTN, VCP*, and *UBQLN2*) as well as sporadic forms of the disease. Finally, we will look to the future of ALS research and how an improved understanding of central mechanisms underpinning proteinopathies might inform research directions and have implications for the development of novel therapeutic approaches.

## Introduction to ALS and ALS-Associated Proteinopathies

**FIGURE 1:**
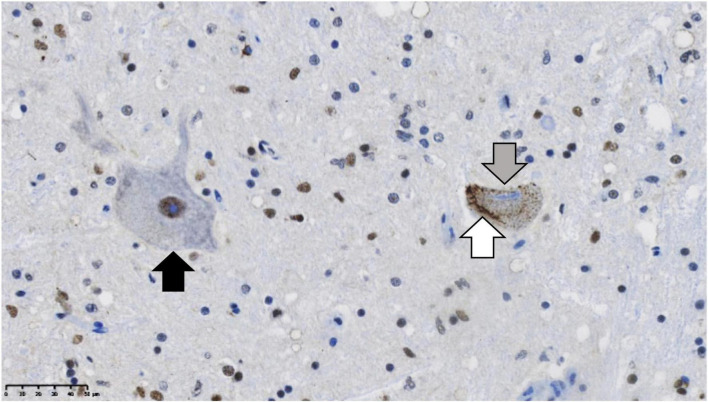
TDP-43 proteinopathy in the post-mortem brain of a sporadic ALS case. Residual motor neurons are immuno-stained for TDP-43 (brown). On the left (black arrow) is a normal motor neuron with the majority of TDP-43 in the nucleus. On the right, there is a diseased motor neuron exhibiting the hallmarks of TDP-43 proteinopathy: formation of filamentous cytoplasmic TDP-43 positive inclusions in the cytoplasm (white arrow), while in contrast, TDP-43 is lost from the nucleus (gray arrow). The nuclei are stained in blue with hematoxylin. Scale bar = 50 μm.

Many neurodegenerative diseases are proteinopathies, the pathological signature of which is the accumulation of protein aggregates. While different neurodegenerative diseases are associated with the accumulation of different proteins, all show the characteristic alteration toward a misfolded, insoluble state. Clinical presentation of neurodegenerative proteinopathies is variable and symptoms often overlap across distinct disorders, and as a result accurate diagnosis can only be carried out post-mortem following immunohistochemical analysis to identify the particular proteinopathy present. This review focuses on proteinopathies associated with amyotrophic lateral sclerosis (ALS) and the ALS-frontotemporal dementia (ALS-FTD) disease spectrum. Inclusions of the trans-active response DNA binding protein (TDP-43) are considered the key proteinopathy in most forms of sporadic and familial ALS, with key familial ALS subtypes ALS-SOD1 and ALS-FUS as the notable exceptions. TDP-43 pathology consists of loss of nuclear TDP-43, neuronal cytoplasmic inclusions (NCIs), dystrophic neurites (DNs), and neuronal intranuclear inclusions (NIIs) and is seen in over 95% of all ALS cases (Neumann et al., 2006). Typical ALS motor neuron TDP-43 mislocalization and pathology can be seen compared to a healthy motor neuron in Figure 1. The unusual lack of TDP-43 positive inclusions in ALS-SOD1 and ALS-FUS provides some insight into the role of specific ALS proteinopathies. ALS can be subdivided into distinct disease types that share common clinical presentations and may share upstream pathogenic mechanisms. This is analogous to the characterization of frontotemporal lobar degeneration (FTLD) as FTLD-TDP or FTLD-tau proteinopathies (Lashley et al., 2015). FTLD-TDP and FTLD-tau are the pathological disease that most commonly cause frontotemporal dementia. Based on the nature of ALS proteinopathies, these subtypes would be ALS-TDP, ALS-SOD1, and ALS-FUS, although as this review will explore, there are many shared mechanisms in common across these groups. While ALS proteinopathies predominantly affect the motor system, there is variable involvement of extra motor brain regions. Furthermore, while the clinical phenotype is related to the extent of neuronal degeneration, there is considerable pathological evidence for the involvement of non-neuronal cell types. The degree of extra motor or glial involvement varies between and within ALS subtypes and will be discussed within this review, for example in ALS-SOD1 there is minimal extra motor involvement compared to ALS-TDP.

### Clinical and Epidemiological Features of ALS

Amyotrophic lateral sclerosis is a fatal neurodegenerative disease, characterized by progressive degeneration of both upper and lower motor neurons of the motor cortex and anterior horn of the spinal cord. The prevalence ranges between 4.1 and 8.4 per 100,000 depending on the geographical region from which the disease is reported, while the peak age of onset ranges from 51–66 years (Longinetti and Fang, 2019). Clinically, patients experience progressive paralysis with approximately two-thirds presenting with a limb-onset phenotype, while the remaining third experience bulbar-onset, presenting with difficulties in speech and subsequently swallowing function. A small fraction of less than 5% of patients present with the respiratory-onset disease (Orrell and Guiloff, 2020). While respiratory onset is associated with the shortest disease duration, ALS generally progresses extremely rapidly with the majority of patients dying from respiratory failure within 5 years of their diagnosis and less than 10% surviving beyond 10 years. ALS is incurable and currently available disease-modifying therapies have only a very modest effect on life expectancy or progression of disability. Current ALS treatment strategies being investigated include a range of drug or antibody based approaches, gene therapy strategies including delivery of antisense oligonucleotides, gene silencing and RNA interference, or supply of neurotrophic factors to support the central nervous system (CNS) (Amado and Davidson, 2021; Corcia et al., 2021; Hautbergue et al., 2021). The diagnosis of ALS is based on tracking clinical symptoms and eliminating other diagnostic possibilities. Family history is also examined although the majority of ALS patients are classed as sporadic cases (sALS), with only some 10% determined to have a familial form of the disease (fALS) (Hobson et al., 2016).

### Major Causative Gene Mutations for ALS

Over 30 ALS-linked gene mutations have now been identified, the majority of which are associated with TDP-43 proteinopathy. A summary of the genetic mutations underlying ALS and ALS/FTD selected for discussion in this review is shown in Table 1. Despite this genetic heterogeneity, four crucial genes account for half of all fALS cases: *C9ORF72* in 40–50% fALS (10% total cases), *TARDBP* (4% fALS; 1% sALS), *SOD1* (20% fALS, 2% sALS), and *FUS* (4% fALS, < 1% sALS). The functions of the proteins encoded by these genes are linked to mitochondria and ATP homeostasis, autophagy, RNA metabolism and protein quality control pathways. Furthermore several ALS associated genes have been identified relating to protein homeostasis: *SQSTM1, OPTN, VCP* and *UBQLN2* (Zou et al., 2017; Chia et al., 2018).

**TABLE 1:** Genes linked to amyotrophic lateral sclerosis (ALS), frontotemporal dementia (FTD), Paget’s bone disease (PBD), inclusion body myopathy (IBM) and multi-system proteinopathy (MSP).

Gene	Chromosome Locus	Gene product	Clinical presentation	Pathological subtype	Typical inclusion subtype	Relevant pathological regions as described in early publications	References
*TARDBP*	1p36	TAR DNA Binding Protein 43	ALS	ALS-TDP	TDP-43-positive NCIs, NIIs, DNs, GCIs as well as Bunina bodies.	Spinal cord, motor cortex, throughout all cortical layers, dentate granule cells of the hippocampus.	Sreedharan et al., 2008
*C9ORF72*	9q21-22	Chromosome 9 open reading frame 72	ALS/FTD	ALS-TDP	Many TDP-43 positive NCIs, some DNs; rare cases with many long corkscrew DNs or with many NCIs, DNs and some NIIs.y TDP-43-negative star-shaped NCIs, DPR positive, ubiquitin-positive and p62-positive.	TDP-43-positive inclusions in hippocampus, including the granule layer of the dentate gyrus, and CA4 sub region; throughout the cerebellar cortex, neocortex, cerebellar granule cells. DPR inclusions throughout the CNS, most reliably in CA3 and CA4 of hippocampus.	DeJesus-Hernandez et al., 2011; Renton et al., 2011; Lashley et al., 2015
*SOD1*	21q22	Superoxide dismutase 1	ALS	ALS-SOD1	SOD1-positive, TDP-43-negative NCIs Neurofilament-positive, SOD1-negative HCIs in some genotypes.	Spinal cord	Rosen et al., 1993
*FUS*	16q12.1-q12.2	Fused in Sarcoma	ALS	ALS-FUS	FUS-positive, TDP-43-negative NCIs and GCIs with highly variable morphology including small bean-shaped and large annular shaped or elongated globular inclusions. Occasional NIIs and DNs	Spinal cord, motor cortex, cerebral cortex, medial temporal lobe, subcortical and brainstem nuclei, basal ganglia.	Vance et al., 2009
*OPTN*	10p13	Optineurin	ALS	ALS-TDP	Inclusions that are positive for optineurin, ubiquitin, and TDP-43 as NCIs including skein-like inclusions and round hyaline inclusions	Spinal cord including corticospinal tracts and anterior horn	Maruyama et al., 2010
*VCP*	9p13	Valosin Containing Protein	ALS ALS/FTD FTD PBD IBM MSP	ALS-TDP	Numerous TDP-43-positive NIIs, DNs and some NCIs., Bunina bodies	Muscle fibers, spinal cord anterior horn, brainstem	Johnson et al., 2010; Mehta et al., 2013; Lashley et al., 2015
*UBQLN2*	Xp11-q12	Ubiquilin 2	ALS ALS/FTD	ALS-TDP	Skein-like or small round TDP-43-positive NCIs, with some larger inclusions of up to 20 μm	Spinal cord, hippocampus including the dentate granule cells and sub regions CA1 and CA3, neuropil and molecular layer of the cerebellar cortex	Deng et al., 2011
*SQSTM1*	5q35.3	Sequestosome 1	ALS-TDP FTD PBD IBM MSP	ALS-TDP	TDP-43- and p62-positive and NCIs including skein-like inclusions, spherical inclusions including Lewy body-like inclusions and basophilic inclusions, small dot-like ubiquitin positive granules and some larger ‘seed like’ NCIs. Thin DNs, numerous Bunina bodies, GCIs	Anterior horn of the spinal cord, cortical layer 2, temporal cortex, entorhinal cortex, granule cells in dentate gyrus of the hippocampus	Arai et al., 2003; Mizuno et al., 2006; Fecto et al., 2011; Teyssou et al., 2013
*TBK1*	12q14.2	TANK-binding kinase 1	ALS FTD	ALS-TDP	Perinuclear TDP-43-positive NCIs and short DNs, NFT-like inclusions, GCIs and some tufted astrocytes	Ventral horn of the cervical and thoraco-lumbar spinal cord, all layers of frontal and temporal neocortex, para-hippocampal gyrus and dentate gyrus, entorhinal cortex, neostriatum and the pallidum	Freischmidt et al., 2015; Van Mossevelde et al., 2016
*CHMP2B*	3p12	Chromatin modifying protein 2B	ALS FTD	ALS-TDP	In FTD cases, TDP-43-negative NCIs, DNs and coiled body type inclusions, GCIs. In cases with an ALS presentation, TDP-43 positive, ubiquitin positive NCIs and GCIs	Throughout the frontal cortex, motor and pre-motor cortex, dentate gyrus of the hippocampus in FTD presentation? In ALS presentation: spinal cord motor neurones, motor cortex	Parkinson et al., 2006; Cox et al., 2010
*hnRNPA1*	12q13.13	Heterogeneous nuclear ribonucleoprotein A1	ALS FTD PBD IBM MSP	ALS-TDP	Rimmed vacuoles and atrophic fibers, cytoplasmic inclusions in muscle. In brain, TDP-43 positive skein-like NCIs	Muscle cells, spinal cord, motor cortex, throughout all cortical layers, dentate granule cells of the hippocampus	Kim et al., 2013
*ANAX11*	10q22.2	Annexin A11	ALS FTD MSP	ALS-TDP	Numerous TDP-43-positive NCIs, including skein-like, filamentous and large tubular shaped inclusions, abundant torpedo-like neuritic structures	Anterior horn of the spinal cord, corticospinal tracts, motor cortex, neuropil, dentate gyrus of the hippocampus, temporal neocortex, occipital lobe	Smith et al., 2017

*Pathological inclusions include neuronal cytoplasmic inclusions (NCIs), dystrophic neurites (DNs), dipeptide-repeat (DPR), hyaline conglomerate inclusions (HCIs), neuronal intranuclear inclusions (NIIs), and glial cytoplasmic inclusions (GCIs).*

#### C9ORF72-Linked ALS

A hexanucleotide G4C2 repeat expansion in the *C9ORF72* gene was identified in 2011 as a major ALS-causing mutation (DeJesus-Hernandez et al., 2011; Renton et al., 2011), accounting for approximately 40% of cases of fALS in European populations although less than 5% in Asian patients (Zou et al., 2017). The discovery of the *C9ORF72* mutation provided an explanation for the link between ALS and frontotemporal dementia (FTD). Characterized by progressive degeneration of the frontal and temporal lobes, FTD is one of the most common forms of early onset dementia affecting 15–22 per 100,000, with up to 40% reporting a family history of the disease (Onyike and Diehl-Schmid, 2013). ALS and FTD are now considered to exist on a single clinical spectrum, with 50% of ALS patients displaying some cognitive or behavioral features to their illness and up to 15% meeting the diagnostic criteria for FTD (Couratier et al., 2017). Clinically, FTD patients present with behavioral or language variants, with the former most commonly associated with the *C9ORCF72* mutation (Lashley et al., 2015).

Proposed pathophysiological mechanisms for the *C9ORF72* mutation include production of dipeptide repeat proteins (DPRs), formation of RNA foci, which are the accumulation of transcribed expanded nucleotide repeats within the nucleus, and haploinsufficiency of the C9ORF72 protein. Despite its location in a non-coding region and lack of start codon, the repeat expansion undergoes an unconventional form of translation known as repeat associated non-AUG (RAN) translation in both sense and antisense directions to produce five neurotoxic DPRs (Castelli et al., 2021). In addition to the typical TDP-43 pathology, C9ORF72-ALS/FTD shows specific DPR pathology from sense and antisense repeat expansion-containing transcripts, comprised of star shaped TDP-43 negative, tau-negative, ubiquitin-positive and p62-positive inclusions throughout the cerebellar cortex, neocortex, and hippocampus. FTLD-TDP pathology is subdivided into types A-D. Type D is exclusively associated with *VCP* mutations (Lashley et al., 2015). Neuronal pathological inclusions are categorized based on their location in the cytoplasm as neuronal cytoplasmic inclusions (NCIs) or within the nucleus as neuronal intranuclear inclusions (NIIs). Dystrophic neurites are processes from the neuronal cell body, which take on a progressively abnormal morphology. This can include taking on aberrant confirmations or becoming shorter and less complex than their healthy counterparts. In C9ORF72-ALS/FTD type B pathology is most common, which features abundant NCIs and some DNs. Type A pathology consists of many NCIs and few DNs, and is less common, while type C with long, corkscrew-like DNs is rarer still (Mahoney et al., 2012). Arginine-rich DPRs have been identified as the most toxic, possibly due to localization, aggregation state, length, variable organelle or cell-specific vulnerability, or a combination of the above. RNA foci sequester and impede the function of many crucial RNA binding proteins (RBPs), with widespread detrimental effects. Additionally, the repeat expansion has a propensity for forming secondary structures, such as G-quadruplexes and R-loops. Secondary structures are used for transcriptional regulation and should have a short lifespan, but unscheduled or increased rates of formation have widespread detrimental effects. These include: damaging genomic integrity, stalling transcriptional machinery and thereby decreasing gene expression, all of which contribute to C9ORF72-ALS/FTD (Reddy et al., 2014; Brèić and Plavec, 2017). Haploinsufficiency arises as carriers of the repeat expansion produce less functional C9ORF72 protein. While *C9ORF72* ortholog knock-outs in *C. elegans* and zebrafish display motor neuron degeneration and motor deficits (Therrien et al., 2013), C9ORF72 knock-out mice show no neurological, behavioral or motor abnormalities but instead have an interesting immune phenotype (Atanasio et al., 2016). In human repeat expansion carriers, individuals homozygous for the *C9ORF72* mutation do not show a more severe disease phenotype compared to heterozygous carriers (Fratta et al., 2013), indicating that while haploinsufficiency may play a role in ALS pathogenesis, it is not the primary driver of the disease.

#### *TARDBP*-Linked ALS

Mutations in the *Trans-active response DNA binding protein* (TDP-43) gene *TARDBP* have been associated with approximately 5% of fALS cases (Shatunov and Al-Chalabi, 2021). TDP-43 plays a complex role in ALS as it is considered the pathological hallmark in nearly all sALS and fALS cases but has also been identified as containing specific disease-causing mutations. Mutations are both loss and gain of function, and those located in the C-terminal are of particular interest due to their association with increased TDP-43 cleavage (Sreedharan et al., 2008). Pathological TDP-43 has a truncated N-terminal, while C-terminal fragments are hyperphosphorylated and ubiquitylated and are detectable in the brains but not spinal cords of ALS patients (Feneberg et al., 2021). The lack of a nuclear localization signal enables a mis-localization from the nucleus and accumulation in the cytoplasm observed as a pathological hallmark in ALS. Once in the cytoplasm TDP-43 is prone to aggregation, with the 35 kDa fragment acting as a seed to recruit full-length TDP-43 to aggregate (Che et al., 2011). *TARDBP* mutations have also been linked to greater propensity for protein aggregation and increased formation of neurotoxic, insoluble TDP-43 species (Guo et al., 2011).

#### *SOD1*-Linked ALS

Superoxide dismutase 1 (SOD1) protein is a widely expressed enzyme whose main function is to scavenge or dismutate superoxide free radicals, and plays an important role in the normal functioning of the mitochondria. *SOD1* was the first causative gene mutation identified for ALS (Rosen et al., 1993). Over 160 *SOD1* variants have now been identified which are associated with 20% of fALS cases via a toxic gain-of-function mechanisms (Shatunov and Al-Chalabi, 2021). ALS-SOD1 is notable in that it lacks the TDP-43 proteinopathy that is considered the pathological hallmark in most cases of ALS (Mackenzie et al., 2007), in addition to lacking behavioral or cognitive symptoms or extra-motor pathological involvement. There are growing calls within the research community to alter the disease classification based on this, with ALS-SOD1 considered a different disease that should be designated apart from cases with TDP-43 proteinopathy. This is consistent with the current pathological classification of frontotemporal lobar degeneration (FTLD), which is classified into FTLD-TDP or FTLD-tau based on the underlying pathology (Lashley et al., 2015).

#### *FUS*-Linked ALS

In 2009 ALS-associated mutations were discovered in *Fused in Sarcoma* (*FUS*) (Kwiatkowski et al., 2009), which encodes a nucleic acid binding protein with roles including transcriptional regulation, and response to DNA damage by recruitment of DNA repair factors (Wang et al., 2013). Associated with a younger age of onset and shorter survival time than ALS-SOD1 cases, over 50 *FUS* mutations have now been identified in association with ALS: predominately missense, but also insertions, deletions, nonsense and splicing mutation variants. Like ALS-SOD1, ALS-FUS cases lack the typical TDP-43 pathology and could therefore also arguably be classed as distinct diseases. *FUS* mutants are exclusively associated with motor abnormalities and not linked to FTD, with FTLD-FUS classed as a sporadic disease not associated with *FUS* mutations (Lashley et al., 2015). FUS is classed as a FET protein, part of a highly conserved and widely expressed family of DNA and RNA binding proteins implicated neurodegenerative diseases (Svetoni et al., 2016). Pathological inclusions seen in FTLD-FUS include other FET proteins and are not seen in ALS-FUS cases (Neumann et al., 2011). Overall, *FUS* mutations account for approximately 5% of fALS cases. The majority of the *FUS* mutations are located in the C-terminal within the nuclear localization signal region, or in the RNA binding RGG motif, consistent with its pathological mis-localization to the cytoplasm and formation of cytoplasmic aggregates (Deng et al., 2010).

Further: The inclusions of FTLD-FUS include other FET proteins (transportin, TAF15 and EWS) and these are not seen in the MND-FUS cases (Neumann et al., 2011) FET proteins TAF15 and EWS are selective markers that distinguish FTLD with FUS pathology from amyotrophic lateral sclerosis with FUS mutations (Neumann et al., 2011).

## Overlapping Pathological Features in ALS Proteinopathies Suggest Common Disease Mechanisms Between ALS-TDP, ALS-SOD1 and ALS-FUS

The existence of ALS-causing mutations in multiple genes presents a substantial challenge for researchers. It is likely that ALS onset, which is usually in the patient’s 5th or 6th decade of life, is the result of many years of accumulating abnormalities that eventually tip over into a disease phenotype. However, at present it remains unclear what initiates this pathogenic process, the most important factors in driving its progression, or at what point different ALS types converge to result in the clinical onset of the diseases, which are broadly similar regardless of underlying genetics. Looking to early stage changes in ALS can help provide clues to identify the most important pathogenic changes. Morphological abnormalities of the distal motor neuron axon are observed as an early stage event in ALS (Fischer et al., 2004), and motor neurons may have specific transport needs rendering them particularly vulnerable to this type of dysfunction. TDP-43 plays a role in microtubule-dependent transport, upon which motor neurons are particularly reliant, by forming cytoplasmic ribonucleoprotein (RNP) granules enabling delivery of mRNA transcripts to the neuromuscular junction and other locations far from the nucleus. RNP granules are large, membraneless structures comprised of RNA and protein, which form large, interconnected networks. This provides dynamic compartments to increase efficiency of biochemical reactions, with functions linked to RNA metabolism and the cellular stress response (An et al., 2021). The TDP-43 C-terminal domain, where most mutations are located, is required for correct RNP granule assembly and ALS mutations are associated with impaired mRNA delivery to distal cellular regions (Alami et al., 2014).

Developing a thorough understanding of the roles of TDP-43 and other key ALS proteins including FUS, SOD1, and C9ORF72 is essential to identify disease mechanisms common across subgroups. Research should focus on the wider network of these proteins, including direct and indirect binding partners and the signaling pathways with which they interact. It is challenging to identify a single starting event that initiates the pathogenic cascade in ALS, or any point of convergence for different ALS subtypes. It is likely that there is no one single common starting event. There are many genes linked to ALS-TDP including *Optineurin, TBK1, TARDBP, C9ORF72*, and *CHMP2B* among others (Nguyen et al., 2018; Shatunov and Al-Chalabi, 2021), and this genetic heterogeneity suggests several possible triggering events leading to a final common pathway of TDP-43 pathology. It is clear from research so far that dysregulation of RNA metabolism plays a key role, although it is unclear if this is a cause or effect of TDP-43 pathology. TDP-43 and FUS have limited overlap in their mRNA binding targets, but overall their interactions cover the vast majority of pathways implicated in ALS (Ling et al., 2013). The interaction network of proteins relevant in ALS is large, complex and interconnected. Polyubiquitin precursor protein ubiquitin-C is one example identified as interconnected with 22 out of 24 proteins known to be causative for ALS. It has been hypothesized that key proteins associated with ALS proteinopathies including TDP-43, FUS, VCP, and hnRNPA1 are examples of ‘essential’ proteins capable of starting a snowballing pathophysiological cascade due to their widespread roles or large numbers of downstream binding partners. Disease onset occurs due to one component in a highly connected network failing and triggering widespread dysfunctions in the regulation of RNA metabolism and protein homeostasis (Mao et al., 2017). Abnormalities include dysregulation of nuclear RNA processing and loss of cytoplasmic RNA binding, resulting in dysfunctional interactions with mRNA targets. In addition to the loss of normal RNA processing, accumulated cytoplasmic inclusions can further sequester RNA-binding proteins (RBPs), or undermine RNA metabolism by impacting adversely on ATP production (Birsa et al., 2020). Mitochondrial dysfunction is an early stage-event linked to accumulation of cytoplasmic proteins and undermines fundamental cellular processes through a failure in energy homeostasis (Smith et al., 2019). Increasingly widespread dysfunction is exacerbated by failures of protein degradation pathways and accumulation of pathological inclusions. The role of these insoluble aggregates has been of great interest to researchers, both in the field of ALS and other neurodegenerative diseases. While they are undoubtedly pathological, there has been substantial debate as to whether inclusions themselves are toxic, if inclusion formation drives disease, or if it is an inevitable result of earlier pathogenic changes. As a broadly consistent ALS disease phenotype is associated with either TDP-43, SOD1, or FUS proteinopathies, it is likely they reflect earlier events which promotes aggregation amongst other neurotoxic effects. To unpick the contribution of aggregates to disease onset and progression, or to try and identify these earlier pathogenic initiating events, we must better understand the conditions under which aggregates are able to form, and at what point their transition toward insolubility becomes irreversible.

### Abnormal Phase Separation Promotes Aggregation in ALS Proteinopathies

It has classically been assumed that in any given cellular compartment that is not subdivided by any membrane, the concentration of various solutes in liquid phase is evenly distributed throughout that compartment. However, in recent years, it has become clear that this is not a valid assumption. Phase separation is an essential biological mechanism used to organize biochemical reactions via membrane-less compartments. Compartments form liquid droplets, which are highly sensitive and associated with abnormal phase transitions and a progressive loss of this delicately organized balance in neurodegenerative diseases (Alberti and Hyman, 2016). Key ALS proteins, including TDP-43, FUS, hnRNPA1, along with numerous other RNA binding proteins, contain a low-complexity prion-like domain. These initiate liquid-liquid phase separation (LLPS) which is the first step in the production of pathological aggregates. Prion-like domains have flexible chains, recurrent aromatic residues and no charge which makes them highly interactive, creating a trade-off between functionality and propensity for aggregation (Harrison and Shorter, 2017). The cell has a tightly regulated network of systems to attempt to prevent these aberrant transitions of proteins with prion-like domains. However, shifts toward more aggregate-prone structures are accompanied by widespread failures in protein degradation systems, and as a result, aggregates that form cannot easily be removed and may act to seed further inclusions. Ribonucleoprotein (RNP) granules have an important role in perpetuating this dysfunction, as they further recruit misfolded, damaged proteins which overwhelmed systems are unable to deal with. In turn, this accumulation promotes even greater conversion to a solid state (Hyman et al., 2014). Additional factors that influence phase transitions are hypothesized to include protein concentration changes, alterations in gene expression, or genomic instability that could alter the delicate balance that maintains RNP granule dynamics and pushes them toward aggregation. Dysfunctional RNA metabolism has been identified as a key mechanism by which RNP granules adopt a more pathological, aggregation prone state. In C9ORF72-ALS/FTD, longer repeat expansions have been linked to greater protein and RNA recruitment to granules, which corresponds to a greater detrimental effect on RNA metabolism (Fay et al., 2017). Longer C9ORF72 repeats provide more sites for potential aberrant molecular interactions, and this may help explain the relationship that exists between longer repeat lengths and an earlier age of disease onset. It also implicates abnormal phase separation events as happening prior to many other pathological events specifically linked to C9ORF72-ALS/FTD such as production of DPRs. Whether this applies to other ALS subtypes remains to be seen. It may be that disrupted RNA interactions are a key mechanism across ALS subtypes, and disease specific mechanisms only contribute at a later point in the disease course (Jain and Vale, 2017).

Fused in Sarcoma has been shown to adopt different solubilities dependent on the concentration of RNP granules, transitioning from a liquid to a solid gel-like state. This concentration-dependent change is influenced by mis-localization and accumulation of FUS in the cytoplasm. This may represent a novel gain of function mechanism in ALS-FUS whereby altering composition and dynamics of FUS compartments occurs due to altering cytoplasmic protein concentrations, increasing the propensity for further aberrant phase transitions. This mechanism may be common across ALS subtypes, involving mis-localization of a variety of proteins depending on the disease type. In *C. elegans*, FUS phase transitions alter other RNP granule components and disrupt normal RNA metabolism (Murakami et al., 2015). Even minor alterations in RNA processing have substantial downstream effects, and once conversion into a more solid state begins, this could trigger further pathological events such as the recruitment of other proteins into early stage aggregates. Mutations accelerate changes in state and altered distribution and behavior of RBPs affects RNP granules, indicating that ALS proteinopathies form due to abnormal phase transitions that occur as a result of RBP mis-localization and associated dysfunction.

Abnormal interactions between TDP-43 low complexity prion-like domains and RNA targets drives formation of pathological inclusions via TDP-43 homo-oligomerization. The RNA recognition motif (RRM) region is implicated, with RNA transcripts acting as a buffer to inhibit phase separation of RNA binding proteins in the nucleus (Maharana et al., 2018). Depleted RNA promotes aggregation of a pathological, abnormal TDP-43 fragment lacking the RRM (Kitamura et al., 2016) and an altered TDP-43 protein-to-transcript ratio is associated with increased aggregation (Mann et al., 2019). Aggregates of TDP-43 are regulated by poly ADP-ribose (PAR) under stress conditions. Tankyrase, a PAR polymerase, is found in neuronal cytoplasmic inclusions in ALS spinal cord tissue and is also elevated in frontotemporal lobar degeneration patient tissue (Tanji et al., 2021). PARylation is a post-translational modification associated with protein degradation, and regulator of tankyrase activity glycogen synthase kinase 3 (GSK3) has also been identified as having a regulatory role in TDP-43 aggregation. Binding between TDP-43 and PAR chains is linked with the former’s mis-localization and promotion of an aggregate-prone state, while tankyrase inhibition suppresses TDP-43 aggregate formation (Tanji et al., 2021). This suggests that there may be multiple paths to motor neuron degeneration via abnormal phase separations, which may help explain the pathological heterogeneity seen in ALS proteinopathies.

Both FUS and TDP-43 show RNA-dependent mis-localization to the cytoplasm (Daigle et al., 2013; Mann et al., 2019), with species not recruited to stress granules vulnerable to forming aggregates. Stress granules are RNA-rich compartments and the dynamism of the interactions between RBPs and their RNA targets helps to maintain the liquidity of the structure and resist conversion to aggregates. Prolonged stress can influence normal stress granule dynamics, causing granules to disintegrate to leave phosphorylated, pathological TDP-43 (McGurk et al., 2018). However, it is the RNA-binding ability that is of central importance: TDP-43 with deficient RNA binding ability is excluded from stress granules and loses its dynamic nature, leading to an aggregation-prone state (Mann et al., 2019). This is contrary to the hypothesis originally proposed that increased propensity for phase separation and aggregation would be linked to increased recruitment to stress granules (Portz et al., 2021). It is still unclear if stress granules have multiple roles, with some preventing aggregate formation, distinct from other stress granules that do seed inclusions. In the latter category it may be that there is a shift in stress granule composition that can occur and promote abnormal phase separation states (Mann et al., 2019). The central importance of RNA binding ability raises questions of how these dysfunctions arise, which may be explained by early stage pathogenic events such as splicing errors. This is supported by evidence from TDP-43 mutant mouse models, which show degeneration of lower motor neurons prior to accumulation of TDP-43 aggregates (Arnold et al., 2013).

### ALS Proteinopathies Coincide With Widespread Alterations of the RNA Metabolism

Several steps in the RNA metabolism are compromised in ALS including transcriptional regulation, alternative splicing, polyadenylation, stabilization and nuclear export of transcripts, axonal transport and microRNA biogenesis. RBPs regulate these processes by forming messenger ribonucleoprotein (mRNP) complexes. Mutations in RBP function are central to ALS proteinopathies. Large numbers of RBPs including TDP-43, FUS, and members of the hnRNP family are used by the cell to regulate the complex series of steps required for RNA processing, maintenance of genomic integrity (Nishida et al., 2017) and protection from DNA damage (Dutertre and Vagner, 2017). Any disruption to RBP function has a large ‘ripple effect’ and once formed, aggregates are able to sequester additional proteins thereby causing increasingly widespread dysfunction. The expression of several thousands genes is affected in ALS affecting up to a third of the transcriptome in ALS-TDP (Arnold et al., 2013; Prudencio et al., 2015), leading non-exhaustively to the alteration of multiple additional biological processes including mitochondrial dysfunction, oxidative and endoplasmic reticulum stresses, altered protein transport, degradation and autophagy, impairments in the nucleocytoplasmic transport of proteins and RNA, excitotoxicity, DNA damage/genomic instability, apoptosis and altered neuronal-astrocyte cross talks.

RNA in the brain undergoes more splicing compared to other tissues (Yeo et al., 2004), underscoring the particular importance of this aspect of RNA metabolism in maintaining neuronal function. Correct splicing of pre-mRNA transcripts relies on complex interactions between many different RBPs and RNAs including miRNAs or long intergenic non-coding RNAs. This is an important mechanism that enables the cell to maintain fine control of gene expression, with almost all genes requiring splicing during transcription. Splicing of mRNA transcripts is governed by spliceosomes, large molecular complexes including small nuclear RNA, and important associated regulator proteins (Rino and Carmo-Fonseca, 2009). Splicing deficits are an important feature of ALS, with RNA splicing dysregulation and altered expression of spliceosome components occurring in motor neurons (Arnold et al., 2013; Highley et al., 2014; Prudencio et al., 2015). Regulatory mechanisms involved in the control of gene expression such as intron retention are also relevant, with abnormal intron retention in mRNA transcripts seen as a unifying feature across different ALS proteinopathies (Luisier et al., 2018). TDP-43 was also reported to suppress the splicing of non-conserved cryptic exons which are located in the intronic regions of genes. Splicing of these cryptic exons into mRNAs in ALS or upon loss of TDP-43 function leads to the loss-of-function of cellular proteins via non-sense-mediated decay and/or alterations of the primary sequences of the encoded proteins (Ling et al., 2015). However, research into splicing alterations often lack functional insight, in that many groups observe alterations in mature mRNA sequences but stop short of determining the functional consequences of these changes. RNA processing is highly sensitive and will be altered in response to any changing physiological conditions in the cell. Neurodegeneration is characterized by an atrophy or a loss of neurons, but also a proliferation of reactive cells such as microglia. This will be reflected in the mRNA transcripts detected in tissue homogenates but may not actually indicate a fundamental issue with splicing and is instead a response to changing pathophysiological conditions. The role of splicing errors in the pathogenesis of sALS and specific fALS subtypes has received little research attention. It is unclear if splicing alterations only impact a selection of patients, or if splicing alterations are actually a late-stage event that can in some circumstances contribute to motor neuron injury but does not represent the upstream drive of ALS pathogenesis.

Another regulatory process that may be relevant across ALS proteinopathies is alternative polyadenylation. In RNA processing, polyadenylation is the process by which a poly adenosine tail is added to an mRNA transcript for the purposes of regulating gene expression. The poly(A) tail can be added at several sites, this is known as alternative polyadenylation (Tian and Manley, 2017). Alternative polyadenylation can result in one gene producing several different distinct 3′ transcripts, and variable UTR length that serve to control translational efficiency and mRNA localization. By this mechanism, the brain contains tissue-specific isoforms of each RNA with 3′UTRs regulated by a number of RBPs with specific mRNA target binding sites (Gupta et al., 2014). Further research is required to establish the role of polyadenylation in different ALS subtypes, and what factors confer vulnerability to motor neurons in particular. The specific ALS associated mutation a patient carries is in general a poor predictor of disease phenotype (Van Mossevelde et al., 2017; Kim et al., 2020) which suggests mechanisms common across disease types that differ in severity based on incompletely understood disease modifiers and environmental factors.

Cell vulnerability to ALS splicing errors may also depend on the ratio of soluble to aggregated RBPs. Splicing errors correlate poorly with pathological inclusions but well with alterations in protein solubility, likely reflecting underlying biochemical changes in a wide array of RBPs which promotes their aggregation (Conlon and Manley, 2017). This suggests ALS subtypes reflect upstream splicing abnormalities affecting one of several key RBPs. In this sense, ALS could be considered a multi proteinopathy defined by which protein is most predominantly affected. It has been hypothesized that patients have a toxicity boundary toward which cells move ever closer as insoluble proteins accumulate and functional RBP levels fall. As this balance shifts, splicing errors are more likely to occur but crucially it is the insolubility and failure of protein degradation systems that are regarded as key pathological events.

#### TDP-43 Proteinopathies

TDP-43 has target binding sites in over 6000 mRNA transcripts and this vast number of interactions means that any abnormalities in this protein result in extremely widespread errors in RNA processing (Xiao et al., 2011). Dysfunctions in splicing are an early stage event associated with *TARDBP* mutations and occur in genes known to be TDP-43 targets (Tollervey et al., 2011), particularly in genes with long intronic sequences (Polymenidou et al., 2011). Notably, these splicing errors take place prior to the major pathological event of mis-localization of TDP-43 from the nucleus (Arnold et al., 2013). The huge number of potentially mis-spliced transcripts as a result of the *TARDBP* mutation may also help explain the widespread dysfunction seen developing later in the disease process. Mis-spliced transcripts are translated to proteins unable to function adequately, undermining entire networks and signaling pathways. As a knock-on effect, the decrease in number of adequate quality transcripts demands that many more proteins, and therefore transcripts, must be produced. This places a larger metabolic burden on the cell. Depleting nuclear TDP-43 with antisense oligonucleotides results in nearly 1000 transcript targets suffering splicing abnormalities in homogenates of mouse brain, particularly in genes encoding synaptic proteins (Polymenidou et al., 2011). Synaptic dysfunction is considered to be an early stage event in ALS and FTD with changes apparent prior to symptom onset (Starr and Sattler, 2018). ALS mutations are expressed throughout the life course, but symptom onset is generally not until well into adulthood. Splicing errors may occur early in neurodevelopment, with abnormalities building throughout life until a disease threshold is reached, or disease onset is triggered by environmental or other factors. Alternatively, splicing errors may only occur later in life, after disease onset. Given the fundamental importance of mRNA processing in the function and survival of the cell, the latter option is arguably more likely, but more research is required to establish where splicing errors fit in the chronology of pathogenic events. If TDP-43 is involved in processing transcripts for other splicing factor proteins, then *TARBDP* mutations have the potential to have an enormous impact far beyond TDP-43’s already plentiful immediate targets. Some indirect effects of TDP-43 dysfunction occurs via its interaction with other RBPs such as hnRNPA1, to which it binds to modulate splicing. TDP-43 nuclear depletion leads to abnormal interactions with hnRNPA1 to promote inclusion of an elongated prion-like exon 7B. This produces an isoform called hnRNPA1B, which is co-localized with TDP-43 pathology and has increased propensity for aggregation and toxicity (Deshaies et al., 2018). This is one example of how altered TDP-43 splicing and mis-localization indirectly leads to a toxic neurodegenerative phenotype via abnormal splicing of its partners such as hnRNPA1. Overall, TDP-43 and hnRNPA1 modulate one third of the transcriptome so resulting RNA metabolism errors are widespread. Mis-splicing events preferentially affect RNA processing genes, leading to altered expression of genes linked with splicing and thereby creating a toxic feedback loop (Highley et al., 2014).

In sALS and fALS with TDP-43 proteinopathies, a type of inclusion called Bunina bodies are also present, but compared to TDP-43 inclusions they have received very little research attention. Bunina bodies are comprised predominantly of human cystatin C (hCC) and located in lower motor neurons (Okamoto et al., 1993). hCC has anti-amyloidogenic effects and regulates autophagy and lysosomal enzymatic activity. The latter occurs through inhibition of proteases including cathepsin B (Wada et al., 2018) and may therefore be linked to the cell’s failure to remove accumulating protein aggregates. Like TDP-43 aggregates, Bunina bodies are present in more than 95% of sALS cases. Mutations of *CST3*, the gene encoding hCC are not linked to ALS, and pathophysiology may instead be linked to deficits in proteins interacting with hCC (Watanabe et al., 2006). One proposed protein that might explain this indirect effect is prosaposin (*PSAP*). hCC binds PSAP, forming a complex which alters its ability to inhibit cathepsin B. Immunohistochemical staining of both hCC and PSAP is decreased in ALS motor neurons containing Bunina bodies (Wada et al., 2018). Mutations in hCC cause hereditary cystatin C amyloid angiopathy (HCCAA), an autosomal dominant disease characterized by brain hemorrhages in young adults, typically leading to death by age 30 (Palsdottir et al., 1988). This is generally at least 20 years before ALS symptoms develop, and there is a lack of research into how the diseases could be linked, if individuals with HCCAA show evidence of pathological TDP-43, and to what extent autophagy is impaired as a result of Bunina body formation. Overall, the extent of pathological hCC and clinical correlations is unknown and requires further research attention.

#### SOD1 Proteinopathies

The role of splicing abnormalities in ALS-*SOD1* is less apparent, with patient derived fibroblasts showing splicing deficits associated with *TARDBP* but not *SOD1* mutations (Highley et al., 2014). Transcriptomics data indicates that intron retention is an early feature in neuronal differentiation found in *SOD1* mutants but not in controls (Luisier et al., 2018), providing evidence of abnormal processing of RNA transcripts. The importance of splicing deficits in other subtypes of ALS and their absence in ALS-*SOD1* provides further support for the concept of genetic heterogeneity in ALS reflecting distinct disease mechanisms associated with ALS-*SOD1* compared to other disease types.

#### FUS Proteinopathies

Splicing deficits and intron retention are also seen in ALS*-FUS* (Luisier et al., 2018), with *FUS* knockouts associated with abnormal splicing in genes associated with motor neuron survival. FUS interacts with spliceosome component U11 snRNP and cytoplasmic aggregates of mutant FUS sequester U11 and U12 into inclusions (Reber et al., 2016). This provides another mechanism relating to RNA metabolism that could be common across ALS subtypes, although it remains to be seen to what extent splicing factors are sequestered by all types of inclusions observed in ALS.

#### C9ORF72-ALS/FTD Proteinopathies

Splicing changes can influence insolubility though interactions with members of the heterogeneous nuclear ribonuclear proteins (hnRNP) family. These proteins include glycine-tyrosine rich intrinsically disordered regions which promote stable complexes with other hnRNPs in a wide, complex network. C9ORF72-ALS/FTD is associated with altered hnRNP H splicing correlating with disease severity, RBP dysfunction and increased insolubility (Conlon et al., 2016). The C9ORF72 repeat expansion is associated with a subtype-specific mechanism of sequestration of proteins by RNA foci as well as dipeptide repeat (DPR) aggregates. Sequestration of hnRNP H results in splicing errors in its RNA targets (Mohagheghi et al., 2016), thereby amplifying the impact of the DPR-induced dysfunction. Other RBPs that are sequestered in C9ORF72-ALS/FTD include ALYREF, SRSF2 and hnRNP-A1 and F (Cooper-Knock et al., 2014), and proteins with RRM or functions linked to splicing and mRNA transport are considered to be vulnerable. Sequestration of transcriptional regulator Pur-α leads to downstream splicing errors. Pur-α interacts with splicing factor SRSF1 (Reddy et al., 2013) which is required for the repeat-containing C9ORF72 mRNA transcripts to exit the nucleus for translation into neurotoxic DPRs (Hautbergue et al., 2017). SRSF1 sequestration leads to increased R-loop formation (Li and Manley, 2005) which in turn increases genomic instability and leads to further sequestration of other RBPs.

### Failures in Protein Degradation Systems Contribute to ALS Proteinopathies

#### Alterations of the Ubiquitin-Proteasome and Autophagy Pathways

The main systems used to process damaged or misfolded proteins are the ubiquitin-proteasome system (UPS), where added poly ubiquitin residues identify proteins tagged for destruction by the proteasome and macro-autophagy. Macro-autophagy degrades protein cargoes within vesicles, via the formation of an autophagosome which fuses with lysosomes. C9ORF72 has been associated with the formation of the autophagosome, while several genes encoding proteins involved in protein degradation including *UBQLN2, SQSTM1, OPTN* and *VCP* are associated with ALS. TDP-43 appears to have a link to autophagy function, with TDP-43 depletion causing autophagy inhibition (Bose et al., 2011). As the cell becomes overwhelmed with mis-localized aggregating proteins, both systems are employed, with TDP-43 aggregates shown to be processed by both the UPS and macro autophagy. Dependent on the level of misfolding, accumulating cytoplasmic TDP-43 is grouped into several species showing differing susceptibility to degeneration, from soluble monomer to insoluble aggregate (Cascella et al., 2017). Thus, Cascella and colleagues found that approximately half of the TDP-43 species were classed as disposable and were split between UPS and autophagy mediated breakdown indicating that these two protein degradation routes play an independent but equally important role in maintaining protein homeostasis.

#### ALS Mutations in Protein Homeostasis Genes Are Associated With Proteinopathies

The importance of protein homeostasis in ALS is highlighted through its associated with mutations in *OPTN, SQSTM1, UBQLN2*, and *VCP* (Chia et al., 2018).

##### Optineurin

Optineurin (OPTN) is a widely expressed protein linked to autophagy, inflammation, and necroptosis. *OPTN* mutations impair these functions and disrupt the neuroprotective network (Markovinovic et al., 2017). ALS associated loss-of-function mutations including exon deletions, frameshifts, non-sense and missense mutations account for up to 4% of fALS and less than 1% of sALS cases (Maruyama et al., 2010; Fifita et al., 2017). *OPTN* mutants are associated with impaired clearance of aggregated proteins (Shen et al., 2015) and damaged mitochondria (Wong and Holzbaur, 2014). Its role in clearing damaged mitochondria is regulated via phosphorylation of OPTN by TANK-binding kinase 1 (TBK1) (Richter et al., 2016). Both *OPTN* and *TBK1* mutants have TDP-43 proteinopathy, and the OPTN protein is incorporated into pathological aggregates present in several neurodegenerative diseases, including the DPR inclusions specific to C9ORF72-ALS/FTD (Bury et al., 2016). Mutations of *TBK1* are also associated with ALS and FTD via a haploinsufficiency mechanism (Freischmidt et al., 2015). It is important to note that ALS-TDP proteinopathies are linked with mutations in genes such as *TBK1* with functions other than protein degradation pathways, indicating that failures in these systems are not the only route to degeneration. In ALS-*SOD1*, TBK1 deletion modulates SOD1 via impairment of protein degradation systems, although results are variable depending on disease stage, suggesting a more complex relationship worthy of further investigation (Brenner et al., 2019).

##### Sequestosome 1

Sequestosome 1 (*SQSTM1*) encodes the p62 protein, which plays a key role in degradation of misfolded or damaged proteins via both proteasome and autophagy pathways. p62 knockout mice have a neurodegenerative phenotype (Ramesh Babu et al., 2008), highlighting the central importance of p62 function and protein degradation pathways more widely in neurodegeneration. Mutations in *SQSTM1* are associated with approximately 2% of fALS and 4% of sALS cases, in addition to FTD and Paget’s bone disease (PBD) (Gennari et al., 2010; Rubino et al., 2012). p62 is a key component in pathological inclusions and almost all inclusions over all ALS subtypes are p62-positive. FUS inclusions are positive for p62 inclusions in post-mortem human tissue (Deng et al., 2010), and p62 co-localizes in the spinal cord of mutant SOD1 mouse models (Gal et al., 2007). A study using post-mortem spinal cord tissue from *SOD1, FUS, C9ORF72*, and *TARDBP*-ALS mutation carriers found p62 positive NCIs in every ALS variant (Keller et al., 2012). This commonality indicates protein degradation failures are likely to be a relatively late-stage event in disease pathogenesis, with different ALS subtypes taking slightly different routes to converge on this common disease pathway.

##### Ubiquilin 2

Mutations in the ubiquilin-2 protein (UBQLN2) have been linked to both fALS and sALS, and in rare occasions to the ALS/FTD spectrum. UBQLN2 is involved in protein degradation, so mutations will disrupt protein clearance mechanisms and result in a build-up of damaged proteins. Inclusions positive for UBQLN2 often co-localize with p62 (Deng et al., 2011), and with TDP-43 in other ALS subtypes such as C9ORF72-ALS (Brettschneider et al., 2012). This provides further evidence for failures of protein degradation pathways as acting as a common mechanism underlying ALS proteinopathies. TDP-43 pathology is present in nearly all ALS cases, indicating that it may be particularly vulnerable to mutations in other ALS associated genes affecting proteostasis.

##### Valosin-Containing Protein

Valosin-containing protein (VCP) is an AAA-ATPase, part of the large family of ATPases associated with diverse cellular activities (AAA). These highly abundant proteins are functionally diverse, with VCP associated with protein homeostasis including degradation, the stress response and apoptosis. It has also been linked to nucleocytoplasmic transport and ATP binding (Song et al., 2015). It forms type D TDP-43 pathology in the neocortex, which consists of many TDP-43- and ubiquitin-positive, tau-negative neuronal intranuclear inclusions and to a lesser extent neuronal cytoplasmic inclusions (Lashley et al., 2015). VCP mutations account for only 1–2% of fALS and have a small role in sALS (Ng et al., 2015), while approximately one third are linked to FTD, Paget’s disease of bone (PDB) and inclusion body myopathy (IBM) (Nalbandian et al., 2011). The ability of VCP mutations to affect multiple tissue types and diverse disease phenotypes reflects the complexity of ALS proteinopathies, with the VCP phenotype likely modulated by genetic and environmental factors. TDP-43 interacts with VCP, which is associated with the formers’ mis-localization and enhanced aggregation (Ritson et al., 2010). TDP-43 may directly impact VCP expression, possibly in a tissue or even individual-specific manner (Abrahao et al., 2016). This is supported by the existence of similar mutations in *hnRNPA1*, *hnRNPA2B1, SQSTM1* that are also associated with TDP-43 inclusions and ALS, FTD, IBM, and PBD (Kim et al., 2013; Bucelli et al., 2015). Recently, VCP mutations have been identified in 5 unrelated Japanese families, where patients present with IBM and ALS/FTD in addition to demyelinating polyneuropathy and ubiquitin pathology (Ando et al., 2021). This suggests that the clinical spectrum of multi system proteinopathy (MSP) which describes this range of conditions may be even wider than previously thought. MSP phenotypes reflect a central pathogenic pathway based on protein degradation and ubiquitin dysfunction that underpins ALS proteinopathies, that do not exclusively affect the central nervous system but instead extend to peripheral dysfunction.

## The Emergence of Multi-System Proteinopathy

Research focus on mutations in protein homeostasis genes such as *VCP* are helping to develop a more accurate picture of ALS proteinopathies through the emergence of the concept of the neurodegenerative disease termed multi system proteinopathy (MSP) (Al-Tahan et al., 2018; Vacchiano et al., 2021). This represents a widening of the disease phenotypes associated with ALS and includes other tissues such as muscle and bone in addition to brain and spinal cord dysfunction. *VCP* mutant patients present with at least two of ALS, FTD, PDB and IBM, and on rare occasions Huntington’s disease (Oskarsson et al., 2015). In a study of 105 muscle biopsy samples derived from patients with *VCP* mutations, ubiquitin and TDP-43 pathology in the form of positive nuclear and cytoplasmic inclusions were also observed (Mehta et al., 2013). This indicates that the traditional characterization of ALS and FTD pathology as confined to brain and spinal cord may be too limited. However, this is an important gap in the literature as many studies investigating ALS proteinopathies have done so in a single cell type, with few making comparisons of pathology between neurons, glia, or muscle and bone. Moreover, most studies have investigated the occurrence of proteinopathies upon overexpression in transfected human cell culture models such as HeLa or HEK cells which are not related to cells found in the CNS.

MSP has also been linked to mutations in other ALS-associated genes, namely *HNRNPA1* and *HNRNPA2B1* (Kim et al., 2013), *SQSTM1* (Bucelli et al., 2015), and a novel missense variant of Annexin A11 (*ANXA11*) (Leoni et al., 2021). *ANXA11* mutations have been linked to ALS in three European families (Smith et al., 2017) and to FTD in one Chinese family (Zhang et al., 2018). Mutations are predominantly located in the N-terminus, which has many sites for protein binding and contains amino acid residues linked to protein folding functions.

Like ALS, IBM features protein misfolding, with proteins previously identified in pathological inclusions generally classed as structural proteins, RBPs, or regulators of protein quality control pathways. As a vesicular trafficking protein, ANXA11 does not fall into any of these categories, however Leoni et al. (2021) identify ANXA11 pathology this indicates that a more widespread characterization of MSP is needed. *ANXA11* mutations are associated with cognitive and behavioral changes which correlate with white matter abnormalities, although not with cortical atrophy. Further investigation, including of MRI data from living patients and post-mortem tissue, will help fully characterize MSP related changes and fully establish the relationship between gene changes, pathology and clinical phenotype in MSP.

### Induction of Mitochondrial Dysfunction

We will now consider the multiple convergent strands of evidence related to mitochondrial dysfunction in ALS. In the subsections that follow we will discuss how TDP-43 induces oxidative stress and mitochondrial dysfunction that contributes to ALS proteinopathies. In addition, we will consider how *FUS* mutations can lead to aberrations of mitochondrial form and function, and how mutant *SOD1*-related impairments alters mitochondrial morphology and reduces respiratory capacity. Finally, we will consider how C9ORF72-ALS/FTD is characterized by mitochondrial damage to mitochondrial respiratory chain complex 1 and the mitochondrial contact site and cristae organising system (MICOS).

#### TDP-43 Induces Oxidative Stress and Mitochondrial Dysfunction That Contributes to ALS Proteinopathies

Despite only accounting for 2% of body weight the human brain requires 20% of the body’s energy resources, and increasingly research into ALS proteinopathies is focusing on mitochondrial dysfunction and subsequent failures of ATP supply. TDP-43 is of particular interest as it binds to mitochondrial tRNAs and transcripts derived from mtDNA in order to maintain mitochondrial homeostasis (Izumikawa et al., 2017). Accumulating cytoplasmic TDP-43, rather than depletion of nuclear TDP-43, has been reported as a good predictor of neuronal death (Barmada et al., 2010). Other studies have shown pathological TDP-43 burden in the spinal cord is associated with a faster disease progression (Cathcart et al., 2021). However, this was not shown for TDP-43 in the motor cortex, and no correlation was observed between TDP-43 burden and the severity of cell loss. TDP-43 nuclear depletion and pathology is loosely correlated with disease severity, leading researchers to look for additional disease modifiers other than inclusion formation. Reduction of TDP-43 led to reductions in processing of mitochondrial RNA transcripts and was associated with impaired mitochondrial function (Izumikawa et al., 2017), while over expression of pathological C-terminal fragments of TDP-43 results in mitochondrial damage and increased mitophagy (Hong et al., 2012). Mutated TDP-43 binds to mitochondrial RNA (mtRNA) transcripts coding for respiratory complex subunits I subunits NADH-ubiquinone oxidoreductase chain 3 and 6 (ND3 and ND6) leading to a disassembly of complex I and a subsequent impairment of ATP supply (Wang et al., 2016). Full-length and pathological truncated TDP-43 fragments are localized to mitochondria in ALS, although it is generally observed in a soluble, un-cleaved and hyperphosphorylated form. There is also evidence of the formation of phosphorylated TDP-43 mitochondrial pre-inclusions (Wang et al., 2016). This mitochondrial localization is associated with abnormal mitochondrial dynamics and morphology, and reductions in mitochondrial length and density, occurring via interactions between TDP-43 and mitophagy regulator protein prohibitin 2 and fusion protein MFN2 (Davis et al., 2018). This mis-localization appears to be central to its toxic effects on mitochondria, as a peptide designed to block this import alleviates neurotoxicity and rescues the disease phenotype in mice, as well as preventing cytoplasmic TDP-43 accumulation (Wang et al., 2017). Cytoplasmic mitochondrial DNA (mtDNA) is released as a result of mitochondrial TDP-43 accumulation and is associated with increased neuroinflammation in ALS patient-derived cell models. This is through the interaction of TDP-43 with cytoplasmic DNA sensor cyclic guanosine monophosphate (GMP)-AMP synthase (cGAS), which can be targeted directly or via signaling partners to prevent the onset of neuroinflammatory changes. cGAS metabolite cGAMP is elevated in ALS patient spinal cord tissue indicating that mtDNA observations in ALS models are consistent with the human disease (Yu et al., 2020).

Increased oxidative stress is a key mechanism through which TDP-43 exerts mitochondrial damage, regulated by phosphorylation of extracellular signal-regulated kinases ERK1/2. In fibroblasts derived from patients with *TARDBP* mutations, abnormal metabolic activity and increases and indices of oxidative stress are associated with the accumulation of cytoplasmic TDP-43 (Romano et al., 2020). TDP-43 cytoplasmic accumulation can be reduced via inhibition of ERK1/2, which triggers TDP-43 to re-enter the nucleus (Romano et al., 2020). This implicates oxidative stress as a possible trigger to cause mis-localization of TDP-43, although it is unclear if alternative kinases can also phosphorylate TDP-43 instead of ERK1/2, or if ERK1/2 is in fact directly phosphorylating TDP-43. More work is needed to clarify the roles of kinases in promoting cytoplasmic TDP-43 localization.

The ample evidence for TDP-43-induced mitochondrial dysfunction is seen also seen *in vivo*, with mitochondrial transport deficits shown in the sciatic nerve of living TDP-43-mutant mice age 45 days, followed by the onset of morphological abnormalities (Magrané et al., 2014). This is consistent with data showing mitochondrial dysfunction in ALS patients, including respiratory chain dysfunction seen in muscle biopsy from sALS and a *TARDBP* mutant patient (Crugnola et al., 2010). sALS and fALS patient fluids show markers of oxidative damage (Bogdanov et al., 2000; Mitsumoto et al., 2008). sALS patients also show alterations in mitochondrial morphology and metabolic disturbances in the anterior horn of the spinal cord (Sasaki and Iwata, 2007). Mitochondrial morphology is also altered and the mitochondrial network fragmented in patient fibroblasts in the presence of a *TARDBP* mutation (Onesto et al., 2016). Together, these data indicates that observations of mitochondria in *in vitro* models of ALS are consistent with *in vivo* models and patient data, providing support for the central role of mitochondrial dysfunction associated with TDP-43 proteinopathy in ALS.

#### FUS Mutants Lead to Abnormal Mitochondria Morphology and Function

Like TDP-43, as an RBP, *FUS* has been shown to alter mitochondrial function through disruption of transcripts for mitochondrial complexes required for ATP production. Mutant *FUS*-induced mitochondrial dysfunction occurs via interactions with ATP synthase β-subunit (ATP5B) to disrupt ATP production, induce loss of mitochondrial cristae and increase mitochondrial fragmentation (Deng et al., 2018). This has been suggested to be one of the earliest pathogenic events in ALS-FUS, and is also observed as an early stage change in mice expressing human FUS prior to any neurodegeneration (So et al., 2018). FUS-positive inclusions show abnormal methylation patterns via interactions with arginine methylase PRMT1 and PRMT8 (Scaramuzzino et al., 2013). Arginine methylation modulates FUS nuclear import, so this abnormal methylation may contribute to the pathological mis-localization (Dormann et al., 2012). Abnormal mitochondrial morphology is observed in FUS-mutant post-mortem human tissue, and corresponding functional defects are associated with an increase in the unfolded protein response (UPR) (Deng et al., 2018). This is likely to be an attempt by the cell to respond to accumulating damaged mitochondrial proteins as a result of FUS induced morphological changes. However, this response may be detrimental, as illustrated by downregulation of ATP5B leading to alleviation of neurodegenerative phenotypes *in vivo*. In ALS, the UPR may be triggered in response to an imbalance in proteins encoded by nuclear or mtDNA due to FUS-induced disruption of ATP synthase and a decrease in mtDNA encoded subunits.

#### Mutant SOD1 Forms Mitochondrial Aggregates and Reduces Respiratory Capacity in Neurons and Skeletal Muscle

As with TDP-43 and FUS, mutant SOD1 forms aggregates within the mitochondria, leading to compromised activity of electron transport chain complexes, decreased respiratory capacity and increased oxidative damage (Deng et al., 2006). Interestingly, human insulin-like growth factor-1 (hIGF-1) has a protective effect in ALS-*SOD1* via upregulation of mitophagy and prevention of apoptosis (Wen et al., 2019). SOD1 induced mitochondrial dysfunction and increased ROS production has been observed in skeletal muscle in an ALS mouse model, even before motor neuron withdrawal from the neuromuscular junction (Xiao et al., 2018). The morphological and functional abnormalities seen in mitochondria derived from SOD1 mutant skeletal muscle are comparable to those seen in motor neurons, including the presence of aggregated SOD1 in the intermembrane space and abnormal depolarization dynamics (Luo et al., 2013). Skeletal muscle mitochondria also show increased expression of cyclophilin D (CypD). CypD regulates the mitochondrial permeability transition pore (mPTP), which might provide a mechanism underpinning accumulation of aggregated SOD1 within the mitochondria (Xiao et al., 2018). It has been hypothesized that increasing SOD1 aggregates promote CypD expression, altering dynamics of the mtPTP, eventually leading to its irreversible opening and creating a pathogenic cascade whereby more SOD1 aggregates can enter the mitochondria. Eventually, mitochondria lose the capacity to respond to ROS production and become permanently damaged and less able to provide a consistent ATP supply. Although research has focused on motor neuron degeneration this finding provides more support for the concept of MSP, characterized by a widening of ALS proteinopathies to include evidence of dysfunction also seen in muscle and bone.

#### C9ORF72-Induced Mitochondrial Damage to MICOS and Respiratory Chain Complex 1 Occurs via Arginine Rich DPRs

Of the three proposed mechanisms for C9ORF72-ALS/FTD disease pathogenesis, haploinsufficiency is the most relevant when considering impairment of mitochondrial function. Haploinsufficiency of C9ORF72 is associated with increased sensitivity to stress (Maharjan et al., 2017) and excitotoxicity, while restoring levels prevents death of patient-derived neurons (Shi et al., 2018). C9ORF72 has been linked to regulation of oxidative phosphorylation and energy homeostasis, via inhibition of protease-dependent degradation of translocase of inner mitochondrial membrane-containing domain 1 (TIMMDC1), which is an essential component of complex 1 in the electron transport chain. Import of C9ORF72 into the mitochondria is a highly conserved evolutionary function, and C9ORF72 haploinsufficiency is associated with a 30–50% reduction in complex I levels (Wang et al., 2021). C9ORF72 acts as a safeguarding factor for complex I and assists with energy buffering capacity, with an estimated 6% of C9ORF72 imported into the inner membrane space. While this is a small proportion of total C9ORF72, the small volume of the inter membrane space means this actually reflects a high concentration. Increasing C9ORF72 expression rescues complex I failures and associated toxicity in C9ORF72-ALS patient derived motor neurons, providing evidence for the importance of C9ORF72 haploinsufficiency (Wang et al., 2021). In C9ORF72-ALS/FTD, haploinsufficiency of the C9ORF72 protein may contribute to disease progression by exacerbating mitochondrial dysfunction.

While impairment in oxidative phosphorylation is seen in *SOD1, FUS*, and *TARDBP*-ALS, a recent report shows that in C9ORF72-ALS only a small reduction in ATP production occurs (Debska-Vielhaber et al., 2021), notably less than in other disease types. This suggests that, unlike other disease subtypes, alternative paths to mitochondrial dysfunction are more relevant for C9ORF72 mutants, such as abnormalities in Ca^2+^ buffering. Ca^2+^ is an essential cell signaling ion and is removed from the cytoplasm by the mitochondria, as high levels are toxic to the cell. In ALS, neurons show high influx of Ca^2+^, increased recovery time and reduced Ca^2+^ buffering ability. In C9ORF72-ALS/FTD patient-derived motor neurons this appears to occur via increased permeability of the Ca^2+^AMPA receptor, and reduced expression of Ca^2+^ importers MICU1 and MICU2, leading to reduced ability of mitochondria to up take cytoplasmic Ca^2+^ (Dafinca et al., 2020). Additionally, the expression of the Ca^2+^ permeable AMPA receptor subunit is upregulated, increasing sensitivity to glutamate and the likelihood of excitotoxicity (Selvaraj et al., 2018). CRISPR/Cas9 can be used to excise the repeat expansion mutation, which reverses these Ca^2+^ homeostasis abnormalities. This suggests that Ca^2+^ buffering may be a subtype-specific pathophysiological mechanism. However, similar or worse abnormalities are also associated with *TARDBP* mutations, with the onset of Ca^2+^ dysregulation occurring prior to TDP-43 accumulation in patient-derived motor neurons (Dafinca et al., 2020). This supports the hypothesis of mitochondrial dysfunction occurring as an early pathogenic event commonly across disease subtypes, but more work in required to establish the role of Ca^2+^ buffering and mutation-specific variations. As with other ALS subtypes, mitochondrial dysfunction is not restricted to neurons. Myocytes containing the *C9ORF72* mutation show formation of TDP-43 aggregates (Lynch et al., 2019). In the light of the emergence of MSP, more extensive research is required to investigate mitochondrial dysfunction in other relevant cell types including bone and muscle.

C9ORF72-ALS/FTD is also linked with some mutation-specific damage to mitochondria through the production of poly GR. Poly GR shows promiscuous binding patterns including to mitochondrial proteins and is linked to an age-dependent increase in oxidative stress (Lopez-Gonzalez et al., 2016). In *Drosophila*, poly GR disrupts the mitochondrial contact site and cristae organising system (MICOS). The MICOS is a dynamic structure which helps to organize and regulate respiration and associated proteins and metabolites and their impairment results in structural and metabolic mitochondrial abnormalities and disrupted ion homeostasis. Targeting ion homeostasis alleviates toxicity, highlighting MICOS disruption as a potential mutation-specific mechanism in C9ORF72-ALS/FTD (Li et al., 2020). Another potential mechanism for poly GR-induced mitochondrial dysfunction is through its interaction with ATP5A1, a component of mitochondrial complex V. In an inducible ALS mouse model, mitochondrial damage is observed starting at only 3 months, including disruption of the inner mitochondrial membrane and loss of cristae, and a decrease in activity of complexes I and V. Reducing poly GR can rescue mitochondrial dysfunction even after disease onset. This provides strong support for mitochondrial dysfunction as one of the earliest and most important pathogenic events in C9ORF72-ALS/FTD (Choi et al., 2019).

#### Astrocytes Contribute to Neuronal Mitochondrial Function in ALS

Astrocytes are the most numerous cell type in the brain and provide significant support for neurons. This includes modulation of synaptic transmission, regulation of blood flow to the brain, maintenance of glutamate levels, and delivery of neuroprotective anti-oxidant molecules (Fernandez-Fernandez et al., 2012; Verkhratsky and Nedergaard, 2018). Astrocytes appear to play a significant role in toxicity in all ALS types (Kok et al., 2021). Therefore failures in astrocyte energy supply and support functions can undermine neuronal survival and contribute to neuronal injury (Magistretti and Allaman, 2015). All major ALS mutations have been associated with astrocyte dysfunction. In C9ORF72-mutant astrocytes a downregulation of anti-oxidants delivered to neurons was observed, including of *SOD1*, which corresponded to increased oxidative stress (Birger et al., 2019). *SOD1* mutant astrocytes themselves show reduced respiratory capacity. Dichloroacetate (DCA) treatment stimulates the pyruvate dehydrogenase complex, alleviating toxicity and improving motor function and survival time in mouse models (Miquel et al., 2012). Conditioned media derived from *SOD1* or *TARDBP* mutant mouse astrocyte cultures results in degeneration of spinal motor neurons linked to oxidative stress (Rojas et al., 2014). Astrocytes provide less support to motor neurons as they age, and this is greatly accelerated in a rodent model overexpressing mutant *SOD1* (Das and Svendsen, 2015). This may provide some insight into the age-related nature of ALS onset.

## Mitochondrial Dysfunction as an Explanation for or a Downstream Consequence of RNA Metabolism and Protein Homeostasis Failures?

Many cellular components and molecular pathways have been implicated in ALS proteinopathies, including endoplasmic reticulum stress, impaired nucleocytoplasmic and axonal transport, DNA damage and genomic instability (Ferraiuolo et al., 2011; Le Gall et al., 2020). DNA damage in particular has previously been implicated in the onset of cellular senescence in the brain, leading to a neurodegenerative phenotype, although recent work has shown no significant association between DNA damage and senescence biomarkers in post-mortem ALS brains. Instead, induction of glial cell senescence was implicated as a contributing factor to cell cycle disruption and early stage disease progression (Vazquez-Villaseñor et al., 2020). Further work is required to explore the full breadth of cellular pathways implicated in ALS, including aberrant interactions between neurons and glia and the onset of senescence and cell cycle dysregulation that underlies the movement from a normal aging phenotype to one of neurodegeneration.

ALS research has focused on RNA metabolism and protein clearance mechanisms largely due to the mutations identified in relevant genes and the fact that the neuropathological hallmark seen in the vast majority of sporadic and mutation-related forms of ALS cases is TDP-43 proteinopathy. TDP-43 proteinopathy can lead to impairments in mitochondrial function, with aggregated TDP-43 also leading to excess release of Ca^2+^, failures in oxidative phosphorylation, increased ROS and apoptosis (Cascella et al., 2019). Accumulation of misfolded proteins exacerbates oxidative stress and results in abnormal oxidative phosphorylation, while antioxidant treatment may help by reducing the stress induced by the misfolded proteins (Debska-Vielhaber et al., 2021). Increased oxidative stress results in TDP-43 recruitment to stress granules (Romano et al., 2020), which may then be pushed toward aggregation prone states.

The question of where mitochondrial dysfunction fits into disease progression is key to understanding ALS proteinopathies. Deficits in protein homeostasis are exacerbated by mitochondrial dysfunction. UPS and autophagy require substantial ATP and if this is unavailable, accumulation of toxic aggregates can occur more readily. Furthermore, high levels of ATP have been linked to maintaining protein solubility and have been shown to help dissolve aggregates (Patel et al., 2017). Dysregulation of metabolism and ATP production have a substantial impact on phase separations. Maintaining separate dynamic compartments is an energy-demanding process and ATP is key for maintaining the activity of helicases and chaperones to regulate formation of RNP granules. Inactivation is linked to a move toward an aggregate-prone structure (Hubstenberger et al., 2013) and altering ATP availability has also been shown to affect stress granule viscosity (Jain et al., 2016). It may be that mitochondrial dysfunction underlies the conversion of dynamic stress granules into aggregates via abnormal phase separations driven by a loss of ATP. Without consistent energy availability the dynamic properties of RNP granules cannot be maintained (Alberti and Hyman, 2016). As mitochondrial function declines and aggregates accumulate it becomes increasingly difficult to maintain normal physical and chemical properties within the cell, leading to the formation of more aggregates which in turn further exacerbates the mitochondrial dysfunction. As an individual ages, mitochondrial function decreases and in the presence of an ALS linked mutation, the metabolic supply cannot meet demand, leading to abnormal phase transitions and failures in protein homeostasis as the cell becomes overwhelmed by misfolded proteins. Disease specific mechanisms may contribute, for example in the *C9ORF72* mutation secondary structures are more prone to forming under conditions of cellular stress, which will worsen the disease phenotype through additional sequestration of RBPs. Poly PR and GR are able to stall ribosomes, blocking protein synthesis, and recruit arginine methylases to induce hypomethylation of a number of endogenous proteins relating to ribosome function and mRNA splicing (Radwan et al., 2020) and Ca^2+^ influx is linked to onset of RAN translation and the integrated stress response (Westergard et al., 2019).

Overall, an intimate relationship between energy metabolism and protein homeostasis deficits are common features across neurodegenerative proteinopathies. In a meta-analysis of 2600 CNS transcriptomics samples from ALS/FTD, Alzheimer’s disease, Lewy body disease and others, these two processes were identified as common neuronal loss-of-function mechanisms. This suggests a single gene expression profile that underpins proteinopathies of very different clinical presentations. This includes upregulation of RNA processing genes, and down regulation of mitochondrial genes linked to the electron transport chain and oxidative phosphorylation (Noori et al., 2021). Advances in “omics” type technology may help uncover more unifying features across proteinopathies although it is essential that transcriptomics data are confirmed to be consistent with protein expression.

## Conclusion

**FIGURE 2:**
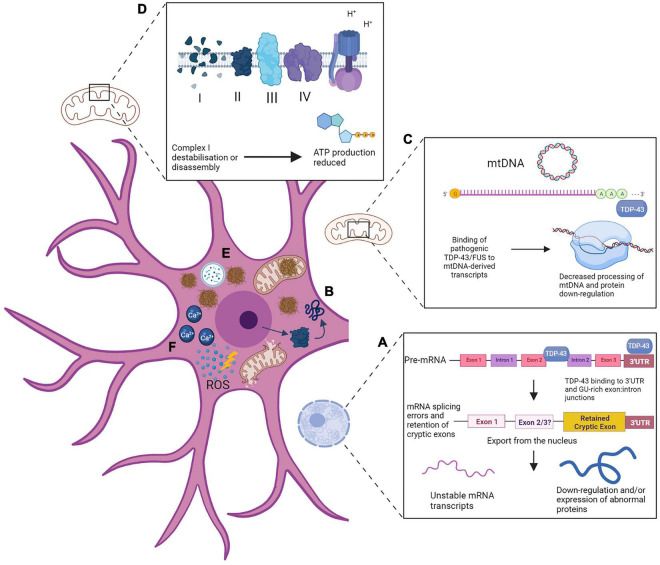
TDP-43 mutations and proteinopathy disrupt multiple biological processes in ALS. While this is depicted in a neuron, effects in glia and other cell types should not be ruled out, but are not included due to a lack of comparable data. **(A)** The pre-mRNA splicing function of TDP-43, one of its well-characterized roles in the RNA metabolism, is compromised in ALS neurons leading to altered alternative splicing as well as insertion of abnormal sequences via splicing of non-coding cryptic exons and downstream widespread los-of-function effects through mRNA instability and potential translation of abnormal/misfolded proteins. **(B)** Mis-localization of proteins such as TDP-43 and FUS from the neuronal nucleus is a major pathological event in ALS. Mis-localized proteins undergo aberrant phase separations, alter stress granule dynamics and develop into insoluble aggregates in the cytoplasm. In some ALS proteinopathies, mis-localized proteins enter neuronal mitochondria, forming pre-inclusions or fully insoluble aggregates, altering mitochondria morphology, dynamics and function. **(C)** Normal mitochondrial function is undermined as pathogenic forms of TDP-43 process mtDNA derived transcripts, resulting in down regulation or misfolding of essential mitochondrial proteins, including those related to the electron transport chain/oxidative phosphorylation, and disrupting mitochondrial homeostasis. **(D)** ALS proteinopathies have been associated with de-stabilization or disassembly of complex I of the electron transport chain, or through interactions with ATP5B thereby reducing ATP production. **(E)** Protein degradation systems become overwhelmed as misfolded proteins and insoluble aggregates accumulate in neurons and glia, while TDP-43 is depleted and ATP supply is compromised. **(F)** Mitochondrial function failing leads to reduced Ca^2 +^ buffering capacity, with an excess of cytoplasmic Ca^2 +^ and an increase in the production of reactive oxygen species triggering excitotoxic damage which will eventually result in mitochondrial breakdown and neuronal degeneration.Created with BioRender.com.

A diagram summarizing the widespread alteration of cellular processes which have been reviewed here is presented in Figure 2. These are depicted in neurons, reflecting the majority of studies of ALS pathologies which rarely attempt to compare molecular mechanisms and are instead confined to one cell type. This is an important area for future research as variations in ALS proteinopathies and associated disease mechanisms, for example in TDP-43 pathology and corresponding splicing deficits, is likely to be more widespread in some types of neuron and glial cells but this is yet to be fully established. From an overall perspective regarding the pathological effects of proteinopathies, the sALS and fALS categorization may be altered to better consider the underlying mechanisms and cellular consequences as the defining feature, giving thus rise to three key types: ALS-*TDP*, ALS-*SOD1* and ALS-*FUS.* Furthermore, we propose that future research should also focus on mechanisms underlying proteinopathies in other related tissues that have so far been largely missed, thereby widening the concept of disease to include multisystem proteinopathy. Ongoing research should treat these three types of ALS as distinct and investigate their common and diverse mechanisms, for example to identify the initial pathogenic events that trigger mitochondrial dysfunction across different ALS subtypes. Regardless of the specific mutation underlying ALS, if any, maintaining RNA metabolism requires consistent high levels of ATP. Neurons are post-mitotic, therefore metabolic activity must be maintained at a high level across a long lifespan. This is particularly true of motor neurons: due to their function and morphology they are vulnerable to mitochondrial defects, which build up over time until a pathogenic cascade is triggered with simultaneous failures of RNA processing and ATP supply (Mao et al., 2017). Eventually, mRNA processing and protein homeostasis will inevitably be undermined by poor energy supply and neurodegeneration will occur.

## Author Contributions

BCB wrote the manuscript with support from GMH and JRH. All authors edited and approved the manuscript.

## Conflict of Interest

GMH, MA, and PJS are inventors on patents granted in the United States (US10801027B2) and Europe (EP3430143B1) for the use of inhibitors of SRSF1 to treat neurodegenerative disorders (WO2017207979A1). The remaining authors declare that the research was conducted in the absence of any commercial or financial relationships that could be construed as a potential conflict of interest.

## Publisher’s Note

All claims expressed in this article are solely those of the authors and do not necessarily represent those of their affiliated organizations, or those of the publisher, the editors and the reviewers. Any product that may be evaluated in this article, or claim that may be made by its manufacturer, is not guaranteed or endorsed by the publisher.
